# Effect of Sterigmatocystin or Aflatoxin Contaminated Feed on Lipid Peroxidation and Glutathione Redox System and Expression of Glutathione Redox System Regulatory Genes in Broiler Chicken

**DOI:** 10.3390/antiox8070201

**Published:** 2019-06-28

**Authors:** Krisztián Balogh, Benjámin Kövesi, Erika Zándoki, Szabina Kulcsár, Zsolt Ancsin, Márta Erdélyi, Csaba Dobolyi, Ildikó Bata-Vidács, Katalin Inotai, András Szekeres, Miklós Mézes, József Kukolya

**Affiliations:** 1 Department of Nutrition, Szent István University, H-2100 Gödöllő, Hungary; 2 Mycotoxins in the Food Chain Research Group, Hungarian Academy of Sciences–Kaposvár University–Szent István University, H-7400 Kaposvár, Hungary; 3 Department of Environmental and Applied Microbiology, Agro-Environmental Research Institute, National Agricultural Research and Innovation Centre (NARIC), H-1022 Budapest, Hungary; 4 Department of Microbiology, Faculty of Science and Informatics, University of Szeged, H-6726 Szeged, Hungary

**Keywords:** sterigmatocystin, broiler chicken, antioxidant defense system, glutathione peroxidase, gene expression

## Abstract

Authors studied the effect of sterigmatocystin from infected corn (STC), purified sterigmatocystin (PSTC), and aflatoxin B1 from infected corn (AFB_1_) on lipid peroxidation and glutathione redox parameters, including the expression of their encoding genes in a sub-chronic (14 days) trial. A total of 144 three-week-old cockerels was divided into four experimental groups (*n* = 36 in each). Control feed was contaminated with STC or PSTC (1590 µg STC/kg or 1570.5 µg STC/kg feed), or with AFB_1_ (149.1 µg AFB_1_/kg feed). Six birds from each group were sampled at day 1, 2, 3, 7 and 14 of mycotoxin exposure. As parameters of lipid peroxidation, conjugated dienes (CD) and trienes (CT) were measured in the liver, while malondialdehyde (MDA) concentration was determined in blood plasma, red blood cell hemolysate and liver. Reduced glutathione (GSH) concentration and glutathione peroxidase (GPx) activity were determined in the same samples, and expression of glutathione peroxidase 4 (*GPX4*), glutathione synthetase (*GSS*) and glutathione reductase (*GSR*) genes was measured by RT-PCR in the liver. STC, PSTC or AFB_1_ caused a slight, but not significant, increase in CD and CT levels; however, in the case of MDA, no increase was found in the liver. Glutathione redox system was activated in the liver by AFB_1_, but less markedly by STC/PSTC. PSTC and AFB_1_ resulted in a higher expression of *GPX4*, while *GSS* expression was down-regulated by AFB_1_ on day 1, but up-regulated by STC on day 2 and by both mycotoxins on day 7. However, on day 14, *GSS* expression was down-regulated by PSTC. Expression of *GSR* was low on day 1 in AFB_1_ and PSTC groups, but later it was up-regulated by AFB_1_. The observed changes regarding gene expression strengthen the hypothesis that the mild oxidative stress, caused by the applied STC doses, activates the glutathione redox system of broiler chickens.

## 1. Introduction

Mycotoxins are secondary metabolites of filamentous fungi, and are toxic to animals and humans. Fungi of *Aspergillus* genus produce several mycotoxins. Aflatoxins—which have more than ten types, the most toxic being AFB_1_—are mainly produced by *Aspergillus flavus* and *Aspergillus parasiticus*. Sterigmatocystin (STC), which has a molecular structure similar to aflatoxins, and acts as an intermediate in the biosynthetic pathway of aflatoxins [1], is mostly produced by *Aspergillus nidulans* and *Aspergillus versicolor* [2]. The presence of STC has been detected in a wide range of crops (e.g., corn, grains, soybean, green coffee bean, nuts), spices, in brewery and dairy products (cheeses), too [3].

Two main target organs of the STC-toxicity are kidneys and liver [4]. The pathological symptoms after STC-exposure are hepatocellular necrosis and hemorrhages in the liver and hyaline degeneration, hemorrhages and tubular necrosis in the kidneys.

STC has also immunomodulatory activity [5,6,7], and mutagenic effect [8,9] has been detected too, in in vivo and in vitro studies. Through induction of chromosomal damages and sister-chromatide exchange [10,11], it leads to cytotoxicity [12,13], inhibition of cell-cycle [14,15] and mitosis [16] as demonstrated in vitro and in vivo.

Metabolic activation of STC is similar to that of AFB_1_—both are metabolized by cytochrome P450 3A4 to a reactive epoxide at the furofuran ring [17]. In the target cells, this active epoxide forms 1,2-dihydro-2-(N^7^-guanyl)-1-hydroxy-STC adducts with the nucleic acids [18,19,20].

The International Agency for Research on Cancer (IARC) classified STC as a 2B carcinogen, which means it is possibly carcinogenic to humans, but a definitive link between human exposure and incidence of cancer has not been proven [21].

Despite the risks, there are still neither regulations nor recommendations for the maximum limits for STC in food or feed, though European Food Safety Authority (EFSA) delivered a scientific opinion about the risk of STC contamination [22].

However, the molecular structures of STC and AFB_1_ are quite similar (both of them are furanocoumarins), the acute toxicity of STC is approximately ten times lower [23].

The toxin explicates its effect through induction of reactive oxygen species (ROS) production during the formation of DNA-adducts [24], which results in oxidative stress in the organism. This may lead to lipid peroxidation, as a secondary mechanism of STC toxicity [25].

The effects of STC on avian species are not fully described—only limited data are available in the case of broiler chickens.

The purpose of present study was to investigate the short-term (up to three days) and longer-term (up to two weeks) effects of naturally, on ground corn substrate produced sterigmatocystin (STC), purified (99%) sterigmatocystin (PSTC) and naturally produced aflatoxin B_1_ (AFB_1_) contaminated feeds on lipid peroxidation, glutathione redox parameters and expression of genes encoding their synthesis or metabolism in some tissues of broiler chickens.

## 2. Materials and Methods

### 2.1. Animals and Experimental Design

A total of 150 one-day-old Cobb 540 cockerels was obtained from a commercial hatchery and were kept on deep litter (room temperature: 26 ± 1 °C, relative humidity: 65 ± 2%). At the age of 21 days, 144 of the chickens were randomly divided into four experimental groups (control, STC, PSTC and AFB_1_) consisting of 36 animals, and each of these groups was divided into two sub-groups (*n* = 18). The basal diet was a commercial broiler grower complete feed (Vitafort Ltd., Dabas, Hungary) free of mycotoxin sequestrates. The nutrient content (on a dry matter basis) of the diet is shown in Table 1. The nutrient content of the diet met the requirements for broiler chickens [26].

### 2.2. Production of Mycotoxin Contaminated Feed

For the three treated groups, the complete feed was contaminated with grits containing STC produced by *Aspergillus creber* 2663, purified (99%) STC (Romer Labs, Tulln, Austria) and grits containing AFB_1_ produced by the high toxin producer *Aspergillus flavus* Zt41 [27], respectively.

To produce sterigmatocystin or aflatoxin B1 contaminated corn, the following method was used. A suspension of 10^8^ conidia/mL was prepared in sterile distilled water from 7-day-old colonies of sterigmatocystin producer *Aspergillus creber* 2663 or aflatoxin producer *Aspergillus flavus* Zt41 grown on PDA (Potato Dextrose Agar, Merck, Darmstadt, Germany) plates. From grits, 2.5 kg portions were transferred into autoclavable plastic bags, 200 mL of distilled water was added, then the bags were autoclaved at 121 °C for 15 min. The grits were left at room temperature to cool. The contents of each bag were transferred to sterile plastic buckets of 10 L (disinfected with UV light and 70% ethanol), and they were each inoculated with 60 mL of *Aspergillus creber* 2663 or *Aspergillus flavus* Zt41 conidium suspension. The inoculum was thoroughly mixed in, and the buckets were covered with sterile canvas material and stored at 26 °C for three weeks. After the incubation period, the samples were sterilized in an autoclave and were dried at 80 °C. The mycotoxin concentrations of the contaminated corn samples were determined by HPLC analytic method: STC concentration was 7.599 mg/kg, AFB_1_ concentration was 4.785 mg/kg. For reaching the targeted mycotoxin contamination in the final feed, toxin containing grits were diluted with toxin-free corn.

In the case of purified sterigmatocystin-contaminated diet, sterigmatocystin was dissolved in absolute ethanol and then sprayed and mixed into the feed.

For setting the final concentrations of the sterigmatocystin contaminated diets, a ten-time correction factor was used, as the proposed toxicity of STC is lower than that of AFB_1_. 

In control diet, the concentrations of STC and AFB_1_ were below the detection limit, while the experimentally contaminated diets contained mycotoxins as follows: STC—1590 µg STC/kg feed; PSTC—1570.5 µg STC/kg feed; AFB_1_—149.1 µg AFB_1_/kg feed.

### 2.3. Quantification of STC and AFB_1_ by HPLC

From the sterigmatocystin or AFB_1_ enriched corn grits and from the control, STC or AFB_1_ contaminated diets, 10 g was transferred into Stomacher bags, and 30 mL methanol was added. The samples were homogenized for 45 s in a Pulsifier instrument (Microgen Bioproducts Ltd., Camberley, UK). The bags were left standing for 24 h in the dark, and then homogenized again for 45 s for thorough extraction. The liquid part of each sample was transferred to 50 mL plastic Falcon tube and centrifuged at 20 °C, 3000 rpm, for 10 min.

For the AFB_1_ measurement, 1 mL of each extract was evaporated and resuspended in 0.4 mL hexane, which was followed by the addition of 0.1 mL trifluoroacetic acid (TFA) and derivatized at 60 °C for 15 min. Then 0.4 mL of water:acetonitril (9:1) was added. After mixing, the lower phase was collected, and 3 µL was applied onto a Prodigy C18 150 × 4.6 mm 5 µm column (Phenomenex, Torrance, PA, USA) in a modular Shimadzu HPLC system equipped with an RF-20A fluorescence detector (Shimadzu Europe, Duisburg, Germany). The separation was carried out at a flow rate of 1 mL/min using isocratic elution (65:35) of water and a mixture of methanol:acetonitril (1:1, *v*/*v*%). The detector wavelengths were 350 nm and 430 nm for the excitation and emission, respectively. For the STC analysis, 5 µL of the metanolic extracts were directly applied onto a Purospher STAR Rp18e 5 µm 250 × 4 mm column (Merck, Darmstadt, Germany) using the above mentioned HPLC system connected to an SPD-10AVP UV-VIS detector (254 nm). The separation was carried out at a flow rate of 0.5 mL/min using gradient elution of water and methanol, which held at 60% for 1 min, then increased to 80% at 9 min, stayed at this rate for 16 min and finally decreased to the starting value till pressure stabilization.

### 2.4. Sampling and Biochemical Determinations

Mycotoxin exposure began at 21 days of age. At the start of the experiment, one randomly selected bird was sampled from each sub-group from which six birds were exterminated as absolute control, resulting 17 birds remaining in each sub-group. Later, six randomly selected birds from each experimental group were sampled on day 1, 2, 3, 7, and 14 of mycotoxin exposure. Blood samples were taken after cervical dislocation. *Post mortem* liver samples were collected, and for gene expression studies, a small portion was put immediately to liquid nitrogen. The whole blood samples were centrifuged (1500 rpm) for 10 min to separate blood plasma. Red blood cell hemolysate was prepared with nine-fold volume of distilled water. All samples were stored at −70 °C until analysis. For the biochemical analyses, a small amount (0.5 g) of the thawed liver samples were homogenized in nine-fold volume of isotonic saline (0.65% *w*/*v* NaCl).

To investigate the initial phase of lipid peroxidation processes, the amount of conjugated dienes (CD) and trienes (CT) in the liver were measured by the ISO 3656:2011 method [28], using trimethyl-pentane and reading the absorption at 232 nm for CD and 268 nm for CT. The terminal phase of lipid peroxidation was followed by the determination of thiobarbituric acid reactive substances (TBARS), based on their color complex formation with 2-thiobarbituric acid [29] and expressed as malondialdehyde, using 1,1,3,3 tetraethoxypropane (Fluka, Buchs, Switzerland) as standard.

Reduced glutathione (GSH) concentration was determined in blood plasma, red blood cell hemolysate and 10,000 g supernatant fraction of 1:9 homogenate of liver in isotonic saline (0.65% *w*/*v* NaCl) by the method described by Rahman et al. [30]. Glutathione-peroxidase (GPx) activity was measured in the same samples as described by Matkovics et al. [31], where the loss of GSH was measured using 5,5′-dithio-bis-2-nitrobenzoic acid (DTNB). GSH content and GPx activity were calculated to the protein content of the samples, which was determined with the biuret method [32] in blood plasma and red blood cell hemolysate, while the method of Lowry et al. [33] was applied for the 10,000 g supernatant fraction of liver homogenates.

### 2.5. RNA Isolation, Reverse Transcriptase and qPCR

RNA extraction was performed from 6–10 mg liver homogenates by NucleoZOL reagent (Macherey-Nagel, Düren, Germany) based on the instructions of the manufacturer. RNA extraction was followed by DNA-se treatment according to the protocol of the manufacturer (Thermo Fisher Scientific, San Jose, CA, USA) to remove any genomic DNA residues. Agarose gel electrophoresis and NanoPhotometer (Implen GmbH, Munich, Germany) measurements were performed to confirm the quantity and integrity of the RNA samples. In addition, only those samples were accepted for further investigation, which had higher ratios of absorption at 260:280 nm than 2.0. For cDNA synthesis, a standard protocol was used with RevertAID Reverse Transcriptase (Thermo Fisher Scientific, San Jose, CA, USA) and random nanomer primer from 1 µg of total RNA. The primers applied for the quantification (Table 2) of the mRNA transcriptional levels of target genes (*GPX4*, *GSS* and *GSR*) and endogenous control gene glyceraldehyde 3-phosphate dehydrogenase (*GAPDH*) were designed with Primer Express 3.0.1 (Thermo Fisher Scientific, San Jose, CA, USA). Endogenous control gene (*GAPDH*) was chosen according to literature data, as it has no known interaction with oxidative stress and mycotoxin exposure in broiler chickens, as it was used as an internal control gene in several other studies [34,35].

Real-time PCR measurements were carried out in duplexes (*GAPDH* and one target gene) applying MGB TaqMan probes (Thermo Fisher Scientific, San Jose, CA, USA) with pooled cDNA template. The pools were formed from equal amounts (100 ng) of cDNA per 6 chicken livers of each sampled group at each sampling point of treatment in five technical replicates. Based on the results of previous experiments, no measurable differences were found if the determination was made from pooled and not from individual samples.

The PCR reaction was performed in PCR tubes (Axygen^®^ Strips for real-time PCR) with StepOne Plus™ real-time PCR systems (Thermo Fisher Scientific, San Jose, CA, USA). For the qPCR, Maxima Probe qPCR Master Mix (1× final concentration) reaction mixture was used (Thermo Fisher Scientific, San Jose, CA, USA). The mix contained 2.5 mM MgCl_2_ and 5 ng cDNA, as well as the primers and dual labelled (6-fluorescein phosphoramidite (FAM) or 2′-chloro-7′phenyl-1,4-dichloro-6-carboxy-fluorescein (VIC) fluorescent die and minor groove binder-non-fluorescent quencher (MGB-NFQ)) TaqMan probes both for target and endogenous control genes in a 12.5 µL final volume per reaction. Furthermore, no-template controls were performed for each PCR measurements.

The dual labelled probes for target and endogenous control genes are listed in Table 3.

The PCR profile was the following: 95 °C for 10 min for pre-amplification denaturation (PAD), and 95 °C 15 sec, 58 °C 30 sec and 72 °C 30 sec for 45 cycles. Both VIC and FAM signals were detected at the end of the extension period. Amplified products were confirmed with gel electrophoresis.

The relative expression level of *GPX4*, *GSS*, *GSR* and endogenous housekeeping control gene, *GAPDH*, was determined by StepOnePlus™ Software v2.2 (Thermo Fisher Scientific, San Jose, CA, USA) using comparative Ct method. The delta Ct values (ΔCt), delta-delta Ct (ΔΔCt) and relative quantification (RQ = 2^−∆∆Ct^) values were calculated according to the formula described by Livak and Schmittgen [36].

### 2.6. Statistical Methods

Statistical analysis of the data was carried out with GraphPad Prism 6.07 software (GraphPad Software, San Diego, CA, USA) after calculating the means and standard deviations (SD).

Normality of parameters’ distribution was tested by Kolmogorov-Smirnov test, and a Barlett test was used to confirm the homogeneity of variance. All data meeting both conditions were compared using one-way ANOVA. Significance of differences between groups was estimated using Tukey-Multiple Comparison post-hoc test (*p* < 0.05). Otherwise, a non-parametric Kruskal-Wallis test with pairwise comparisons was used (*p* < 0.05). Data are presented as mean ± standard deviation (SD).

### 2.7. Ethical Statement

The experiment was carried out according to the Hungarian Animal Protection Act, in compliance with the EU rules. The experimental protocol was authorized by the Department of Food Chain Safety, Land Register, Plant and Soil Protection and Forestry of the Pest County Government Office (Hungary) with a permission number PE/EA/1964-7/2017.

## 3. Results

The average feed consumption during the two weeks long experiment was 136.2 g/bird in the control group. In mycotoxin treated groups, slightly lower feed intake was measured than in control. In decreasing order: STC 134.0 g/bird (−1.58%); PSTC 128.0 g/bird (−6.01%); AFB_1_ 125.7 g/bird (−7.70%). The lower feed intake resulted significantly (*p* < 0.05) lower body weight in the STC group as compared to control on the seventh day of mycotoxin exposure. On the same sampling day, the average body weight of the STC group was significantly (*p* < 0.01) lower than of the AFB_1_ group (data not shown).

Absolute liver weight (g) was significantly (*p* < 0.05) lower on the second day of mycotoxin exposure in the PSTC group as compared to the control. On the seventh day, the absolute liver weights of the STC treated chickens were significantly (*p* < 0.001) lower than that of the AFB_1_ treated birds. The calculated relative liver weight (g/100 g body weight) in the STC group was significantly (*p* < 0.05) lower at the last sampling (14th day) as compared to the control group (data not shown).

Amounts of conjugated dienes (CD) and trienes (CT) were measured in the liver as markers of the initial phase of lipid peroxidation, while malondialdehyde (MDA) concentration as a meta-stable terminal phase marker was determined in blood plasma, red blood cell hemolysate and liver. Both applied doses of STC/PSTC and AFB_1_ revealed pro-oxidant effect, and caused a marked, but not significant, increase in CT and CD, but not in MDA levels in the liver (Table 4).

The increase of free radical formation in the liver activated the glutathione redox system, as it was supported by the slightly elevated GSH concentration at both short (1st day) and long-term (14th day) AFB_1_ exposure compared to the control. On the second day, AFB_1_ caused significantly (*p* < 0.05) higher GSH concentration in the liver than PSTC treatment (Table 5).

GPx activity of liver showed similar changes as its co-substrate (GSH), resulting in higher values at the start (first and second day) and at the end (14th day) of AFB_1_ treatment compared to control. On the second day of exposure, PSTC treatment caused significantly (*p* < 0.05) lower GPx activity than in control and in AFB_1_ treated groups (Table 5).

In blood plasma, AFB_1_ exposure resulted significantly (*p* < 0.05) lower GSH concentration as compared to control at the last sampling (6.99 ± 1.28 vs. 8.74 ± 1.20 µmol/g protein). GPx activity showed similar, but not significant changes as observed in its co-substrate, GSH (data not shown).

The two-week-long mycotoxin exposure did not result in significant alterations in MDA concentration, amount (GSH) and activity (GPx) of the glutathione redox system in red blood cell hemolysates (data not shown).

The gene expression of *GPX4* was significantly lower on the first day of mycotoxin exposure in AFB_1_ group than in the control and STC groups. Later, purified STC (day 2 and 7) and aflatoxin treatments (day 2) resulted in significantly higher expression of *GPX4* gene as compared to the control. At day 2, in AFB_1_ group, expression of *GPX4* gene was significantly higher than in control, while at days 7 and 14 the gene expression was significantly higher in AFB_1_ group than in the control group (Table 6).

Similar to the findings in the case of *GPX4* on the first day of mycotoxin exposure, gene expression of *GSS* was also significantly lower in AFB_1_ group than in the control and STC groups.

On the second day, elevated *GSS* gene expression was observed in the STC group compared to the control. On the seventh day, the expression of the *GSS* gene exceeded the control in all treated groups. A week later, the lowest *GSS* expression was measured in the PSTC group, which was significantly different from the others (Table 6).

The gene expression of *GSR* was significantly lower on the first day of mycotoxin exposure in AFB_1_ and in PSTC groups than in the control and STC groups. On the second and seventh day of mycotoxin exposure, aflatoxin treatment significantly increased the expression of *GSR* gene compared to the control group. On the third and seventh days, the expression of *GSR* gene in the PSTC group was significantly higher than in control, while on the 14th day, STC treatment caused significantly elevated expression (Table 6).

## 4. Discussion

The applied dose (149.1 µg AFB_1_/kg feed) of aflatoxin in the feed of broiler chickens in this two-week-long feeding trial was 7.5-times higher than the regulatory limit in the EU (Commission Regulation 574/2011). It caused some alterations in production parameters (e.g., lower feed intake), had a rapid pro-oxidant effect and caused elevations in the initial, but not in the terminal phase parameters of lipid peroxidation processes in the liver, and also in the amount and activity of the glutathione redox system. The liver is the primary site of the reduced glutathione biosynthesis [37], which tripeptide—as co-substrate of selenium-dependent glutathione peroxidases and glutathione-S-transferases—has an important role in protection against the reactive oxygen species (ROS). However, Karaman et al. [38] reported depletion of GSH concentration in the liver of broilers as the effect of aflatoxin exposure, but, in the present study, no such changes were observed—possibly due to the lower dose and shorter duration of exposure. Regarding GPx activity, the literature reported marked reduction in the case of AFB_1_ exposure [39], but in this trial, GPx activity did not decrease significantly in the liver, though a slight increase was observed, which was possibly caused by the lower dose and shorter duration of exposure. Thus, increasing ROS formation in the cells activated the enzymes involved in the biological antioxidant system and also their gene expression. Hepatic gene expression of antioxidant enzymes, such as superoxide dismutase (SOD), GPx and glutathione S-transferase (GST) were affected (mostly down-regulated) in chickens fed with diets containing AFB_1_ [34,40]. In this trial, significant down-regulation of *GPX4* gene was also observed a day after the start of AFB_1_ exposure, but later, at day 2 and day 14, up-regulation was found, suggesting an effective activation of the glutathione redox system as a response to mild oxidative stress. In the antioxidant defense system of birds, glutathione peroxidase 4 (GPX4) is more important [41] than in the case of mammals, where GPX1 plays the major role [42], which is also supported by our findings. Glutathione synthetase (GSS) catalyses the reaction of gamma-glutamylcysteine with glycine [37] to form reduced glutathione (GSH). Gene expression encoding this enzyme, involved in the two-step synthesis of GSH, showed dual response to the AFB_1_ exposure. A rapid down-regulation (day 1) as an early effect of oxidative stress was followed by up-regulation (day 7), suggesting effective response, but turned again to down-regulation at the end of the trial (day 14), possibly due to effective antioxidant response, which was supported by the lack of increase in the termination phase marker of lipid peroxidation, malondialdehyde content, in the liver. Glutathione reductase (GSR) catalyses the reduction of glutathione disulphide (GSSG) to reduced glutathione (GSH), maintaining the reducing environment and the sulfhydryl pool of cells [37]. Gene expression changes of *GSR* as a result of AFB_1_ exposure was similar to *GSS* described earlier, a rapid down-regulation (day 1) was followed by up-regulation (at days 2 and 7), which also suggests an adequate antioxidant response to oxidative stress after an early inhibitory phase regarding gene expression.

Sterigmatocysin is carcinogenic, although its carcinogenicity is about 10–100 times less than that of AFB_1_ in test animals [43]. The intake of STC contaminated feeds at the applied concentration, which was ten times higher than the EU regulatory limit for aflatoxin B1, caused lower body weight and reduced absolute and relative liver weights compared to the control or aflatoxin treated broilers, which means that the dose applied has a toxic effect in chicken. The literature is very scarce in connection with the effect of STC on the biological antioxidant system of avian species. The main site of its biotransformation is the liver, in which phase I enzymes (cytochrome P450 superfamily) and phase II processes are involved. The latter uses the glutathione pool of this tissue to form GSH adduct of monooxygenated STC to increase its excretion through the bile juice. In a long-term experiment with rats, STC exposure resulted in the generation of oxygen free radicals, which caused lipid peroxidation, namely elevated MDA concentration. This result was explained by the depletion of antioxidant defense [25]. In a recent experiment with broilers, slight elevation was observed in initial phase markers of lipid peroxidation, but the termination phase marker and the amount and activity of the glutathione redox system did not increase, which can be explained by the fact that the level of oxidative stress did not reach the critical level for its significant activation. These results suggested that STC has a lower oxidative stress-inducing effect than the predicted one-tenth of AFB_1_. There were some differences in the effects regarding protein level by naturally occurring and purified STC, which were probably caused by some other non-identified metabolites in the naturally occurring preparation. Purified STC caused significantly lower GPx activity in the liver at day 2, which was probably caused by the lower GSH concentration at the same sampling-time, which is essential for GPx activity [44]. In gene expression levels, the purified form of STC caused up-regulation of *GPX4* at days 2 and 7, and naturally occurring STC at day 14, as compared to control, which suggests activation of antioxidant gene cluster even in the case of mild oxidative stress, but it requires different periods of exposure. Regarding *GSS* expression, the pattern of changes was similar to AFB_1_ in the case of both applied forms of STC. A quick down-regulation (day 1) was followed by up-regulation (day 7), which turned again to down-regulation at the end of this trial (day 14), suggesting the response to mild oxidative stress up to day 7. In *GSR* expression, the two applied forms of STC resulted in dissimilar changes. At days 1 and 7, the purified form of STC caused similar down-regulation as AFB_1_ did, but on corn grits produced form caused the opposite effect at every sampling, possibly due to the presence of some non-identified metabolites in the naturally occurring STC.

## 5. Conclusions

In conclusion, the results revealed that STC caused mild oxidative stress in chicken liver, and activated the glutathione redox system, as suggested by the changes regarding the level of gene expression, which did not manifest at the protein level, as well. The results also showed that the oxidative-stress induction activity of STC is lower than one-tenth of AFB_1_, as predicted according to other toxicity data.

## Figures and Tables

**Table 1:** Nutrient content of the broiler grower complete feed.

Metabolisable Energy (ME)	10.69 MJ/kg
Crude protein	19.34%
Crude fiber	4.10%
Ether extract	2.90%
Crude ash	7.40%
Calcium (Ca)	1.02%
Phosphorus (P)	0.70%
Sodium (Na)	0.15%
Lysine (Lys)	0.95%
Methionine (Met)	0.45%
Vitamin A	10,050 IU/kg
Vitamin D_3_	3015 IU/kg
Vitamin E	34 mg/kg

**Table 2:** The used real-time PCR primers of target and endogenous control genes.

Gene	Forward Primer 5′–3′	Reverse Primer 5′–3′	Accession Nr.
*GAPDH*	TGACCTGCCGTCTGGAGAAA	TGTGTATCCTAGGATGCCCTTCAG	NM_204305.1
*GPX4*	AGTGCCATCAAGTGGAACTTCAC	TTCAAGGCAGGCCGTCAT	NM_001346448.1
*GSS*	GTACTCACTGGATGTGGGTGAAGA	CGGCTCGATCTTGTCCATCAG	XM_425692.6
*GSR*	CCACCAGAAAGGGGATCTACG	ACAGAGATGGCTTCATCTTCAGTG	XM_015276627.2

**Table 3:** Dual labelled probes for target and endogenous control genes. Minor groove binder-non-fluorescent quencher (MGB-NFQ) quencher was used in all probes.

Gene	MGM-NFQ Dual Labelled Probe 5′–3′	Fluorescent Dye
*GAPDH*	CCAGCCAAGTATGATGAT	VIC
*GPX4*	CAGCCCAATGGAG	FAM
*GSS*	AGGAGGGAACAACCTG	FAM
*GSR*	CTGGCACTTCGGCTC	FAM

**Table 4:** Effect of sterigmatocystin and aflatoxin on conjugated dienes (CD), trienes (CT) and thiobarbituric acid reactive substances (TBARS) contents in the liver of broiler chickens (mean ± SD, *n* = 6).

**Conjugated Dienes (OD 232 nm)**				
**Samplings**	**Control**	**STC**	**PSTC**	**AFB_1_**
0 h	0.315 ± 0.027			
Day 1	0.261 ± 0.026	0.279 ± 0.028	0.267 ± 0.041	0.296 ± 0.024
Day 2	0.293 ± 0.043	0.334 ± 0.089	0.323 ± 0.059	0.297 ± 0.039
Day 3	0.295 ± 0.041	0.295 ± 0.047	0.304 ± 0.031	0.326 ± 0.023
Day 7	0.313 ± 0.028	0.317 ± 0.036	0.307 ± 0.040	0.320 ± 0.023
Day 14	0.311 ± 0.049	0.290 ± 0.026	0.292 ± 0.029	0.305 ± 0.017
**Conjugated Trienes (OD 268 nm)**				
**Samplings**	**Control**	**STC**	**PSTC**	**AFB_1_**
0 h	0.168 ± 0.016			
Day 1	0.135 ± 0.012	0.147 ± 0.015	0.138 ± 0.018	0.153 ± 0.010
Day 2	0.145 ± 0.015	0.166 ± 0.033	0.159 ± 0.027	0.157 ± 0.022
Day 3	0.156 ± 0.021	0.151 ± 0.017	0.152 ± 0.012	0.158 ± 0.014
Day 7	0.165 ± 0.012	0.161 ± 0.019	0.157 ± 0.019	0.161 ± 0.012
Day 14	0.155 ± 0.007	0.158 ± 0.012	0.162 ± 0.014	0.164 ± 0.012
**Thiobarbituric Acid Reactive Substances (Malondialdehyde, µmol/g Wet Weight)**				
**Samplings**	**Control**	**STC**	**PSTC**	**AFB_1_**
0 h	19.85 ± 6.47			
Day 1	30.45 ± 12.98	38.99 ± 16.25	26.31 ± 3.27	31.06 ± 4.61
Day 2	25.96 ± 8.44	24.49 ± 6.97	22.18 ± 3.91	24.11 ± 2.78
Day 3	17.73 ± 3.53	14.95 ± 6.97	16.19 ± 4.73	18.62 ± 6.99
Day 7	28.78 ± 2.68	25.24 ± 5.61	28.33 ± 6.06	23.20 ± 1.01
Day 14	18.53 ± 3.13	19.91 ± 2.78	20.74 ± 4.10	23.39 ± 3.57

STC—1590 µg STC/kg feed; PSTC—1570.5 µg STC/kg feed; AFB_1_—149.1 µg AFB_1_/kg feed.

**Table 5:** Effect of sterigmatocystin or aflatoxin on reduced glutathione (GSH) concentration and glutathione peroxidase (GPx) activity in the liver of broiler chickens (mean ± SD; *n* = 6).

**GSH (µmol/g Protein)**				
**Samplings**	**Control**	**STC**	**PSTC**	**AFB_1_**
0 h	3.47 ± 0.38			
Day 1	4.84 ± 0.98	4.56 ± 0.79	5.03 ± 0.74	5.17 ± 1.44
Day 2	4.49 ^ab^ ± 0.73	4.46 ^ab^ ± 0.55	3.63 ^a^ ± 0.83	4.79 ^b^ ± 0.41
Day 3	4.81 ± 0.79	3.99 ± 1.42	3.90 ± 1.04	4.66 ± 2.23
Day 7	7.41 ± 0.55	6.58 ± 0.41	7.69 ± 1.32	7.27 ± 0.95
Day 14	5.34 ± 0.42	4.97 ± 0.75	5.95 ± 0.66	6.89 ± 3.11
**GPx (U/g Protein)**				
**Samplings**	**Control**	**STC**	**PSTC**	**AFB_1_**
0 h	3.37 ± 0.29			
Day 1	4.68 ± 1.00	4.41 ± 0.71	5.18 ± 0.91	5.46 ± 0.71
Day 2	3.97 ^b^ ± 0.46	3.62 ^ab^ ± 0.35	3.06 ^a^ ± 0.62	4.10 ^b^ ± 0.56
Day 3	4.48 ± 0.99	4.04 ± 1.32	3.96 ± 0.87	4.34 ± 1.99
Day 7	6.33 ± 0.72	6.16 ± 0.42	6.84 ± 1.32	6.01 ± 0.39
Day 14	5.20 ± 0.36	4.66 ± 0.93	5.58 ± 0.62	6.27 ± 2.73

^a,b^ Different superscripts in the same rows mean significant difference at *p* < 0.05 level. STC—1590 µg STC/kg feed; PSTC—1570.5 µg STC/kg feed; AFB_1_—149.1 µg AFB_1_/kg feed.

**Table 6:** Effect of sterigmatocystin and aflatoxin on relative gene expression of glutathione peroxidase 4 (*GPX4*), glutathione synthetase (*GSS*) and glutathione reductase (*GSR*) in the liver of broiler chickens (mean ± SD; *n* = 6, in a pool, equal amount of cDNA).

**Gene Expression of *GPX4***				
**Samplings**	**Control**	**STC**	**PSTC**	**AFB_1_**
0 h	1.00 ± 0.05			
Day 1	0.57 ^bc^ ± 0.02	0.63 ^c^ ± 0.03	0.52 ^ab^ ± 0.02	0.50 ^a^ ± 0.04
Day 2	0.57 ^a^ ± 0.03	0.59 ^ab^ ± 0.01	0.62 ^b^ ± 0.03	0.63 ^b^ ± 0.02
Day 3	0.67 ^a^ ± 0.03	0.62 ^a^ ± 0.04	0.71 ^a^ ± 0.07	0.60 ^a^ ± 0.02
Day 7	0.51 ^ab^ ± 0.04	0.48 ^a^ ± 0.03	0.67 ^c^ ± 0.06	0.59 ^b^ ± 0.04
Day 14	0.48 ^a^ ± 0.03	0.57 ^c^ ± 0.05	0.50 ^ab^ ± 0.03	0.55 ^bc^ ± 0.03
**Gene Expression of *GSS***				
**Samplings**	**Control**	**STC**	**PSTC**	**AFB_1_**
0 h	1.00 ± 0.02			
Day 1	0.55 ^d^ ± 0.04	0.46 ^c^ ± 0.02	0.38 ^ab^ ± 0.05	0.34 ^a^ ± 0.05
Day 2	0.45 ^a^ ± 0.05	0.57 ^b^ ± 0.03	0.52 ^ab^ ± 0.05	0.54 ^a^ ± 0.06
Day 3	0.66 ^ab^ ± 0.05	0.61 ^ab^ ± 0.08	0.60 ^a^ ± 0.08	0.70 ^b^ ± 0.05
Day 7	0.41 ^a^ ± 0.04	0.50 ^b^ ± 0.05	0.70 ^c^ ± 0.06	1.04 ^d^ ± 0.02
Day 14	1.03 ^c^ ± 0.06	0.93 ^b^ ± 0.05	0.74 ^a^ ± 0.09	0.91 ^b^ ± 0.04
**Gene Expression of *GSR***				
**Samplings**	**Control**	**STC**	**PSTC**	**AFB_1_**
0 h	1.00 ± 0.03			
Day 1	0.43 ^b^ ± 0.06	0.41 ^b^ ± 0.05	0.28 ^a^ ± 0.03	0.24 ^a^ ± 0.02
Day 2	0.36 ^a^ ± 0.05	0.40 ^ab^ ± 0.06	0.41 ^ab^ ± 0.05	0.46 ^b^ ± 0.04
Day 3	0.47 ^a^ ± 0.04	0.50 ^a^ ± 0.07	0.68 ^b^ ± 0.10	0.43 ^a^ ± 0.03
Day 7	0.42 ^a^ ± 0.03	0.39 ^a^ ± 0.07	0.55 ^b^ ± 0.07	0.84 ^c^ ± 0.05
Day 14	0.40 ^a^ ± 0.04	0.64 ^b^ ± 0.08	0.41 ^a^ ± 0.06	0.44 ^a^ ± 0.08

^a,b^ Different superscripts in the same rows mean significant difference at *p* < 0.05 level. STC—1590 µg STC/kg feed; PSTC—1570.5 µg STC/kg feed; AFB_1_—149.1 µg AFB_1_/kg feed.
